# Human Papillomavirus Infection, p16^INK4a^ Expression and Genetic Alterations in Vietnamese Cervical Neuroendocrine Cancer

**DOI:** 10.21315/mjms2019.26.5.15

**Published:** 2019-11-04

**Authors:** To Van Ta, Quang Ngoc Nguyen, Van-Long Truong, Toan Trung Tran, Hung Phi Nguyen, Linh Dieu Vuong

**Affiliations:** 1 Pathology and Molecular Biology Center, National Cancer Hospital K, Thanh Tri, Hanoi, Vietnam; 2 Department of Smart Food and Drug, College of BNIT, Inje University, Gimhae, Korea

**Keywords:** HPV, p16^INK4a^, p53 mutation, NRAS mutation, neuroendocrine cervical cancer

## Abstract

Neuroendocrine cervical cancer is a rare subtype of cervical cancer with a highly aggressive malignancy. This study was conducted to analyse the human papillomavirus (HPV) infection and molecular abnormalities in Vietnamese neuroendocrine carcinomas of the uterine cervix. HPV genotyping and p53 mutations were examined using polymerase chain reaction (PCR)-based direct sequencing. Mutations of epidermal growth factor receptor (EGFR), Kirsten rat sarcoma (KRAS), neuroblastoma RAS viral oncogene homolog (NRAS) and v-Raf murine sarcoma viral oncogene homolog B (BRAF) were identified using commercial kits. Four high-risk HPV genotypes were identified in 26 (86.7%) out of a total of 30 tumours. The prevalence of HPV 16, 18, 31 and 45 was 20.0%, 50.0%, 20.0% and 36.7%, respectively. Overexpression of p16^INK4a^ was observed in 93.3% of cases and was significantly correlated with high-risk HPV infections. Furthermore, p53 and NRAS mutations were detected in five (16.7%) and one (3.3%) cases, respectively, whereas no EGFR, KRAS or BRAF mutations were observed. These results demonstrate that high-risk HPV infection may be an important oncogenic factor for the development and progression of cervical neuroendocrine carcinoma.

## Introduction

Cervical cancer is the second most common type of cancer and the fourth leading cause of cancer mortality in women worldwide, with an estimated 530,000 cases per year and a disproportionate impact on developing countries (1). Cervical neuroendocrine cancer is a rare epithelial cancer, accounting for 0.5%–3% of all cervical carcinomas (2). In contrast to other cervical cancers, neuroendocrine cancer of the cervix is a highly aggressive malignancy with a poor prognosis in the early stages. Cervical neuroendocrine carcinoma encompasses several histological subtypes, including small cell neuroendocrine carcinoma (SCNC), large cell neuroendocrine carcinoma (LCNC), typical carcinoid tumour and atypical carcinoid tumour (3). Of these four subtypes, SCNC and LCNC are the most aggressive phenotypes, representing the majority of neuroendocrine cancers of the cervix. In addition, neuroendocrine carcinoma of the cervix has a strong tendency toward lymph node metastasis and lymph-vascular space invasion, which are signs of poor prognosis found in most malignant tumours (3).

Human papillomavirus (HPV) infection plays a critical role in the pathogenesis of cervical cancer. HPV can be categorised into high-risk and low-risk HPV types based on each type’s correlation with cervical cancer and precursor lesions. High-risk HPV genotypes—such as HPV 16, 18, 31, 45, 56 and 68—are detected in 99% of cervical cancers and approximately 70% of cervical cancers are related to the presence of HPV 16 and 18 (4). Especially, HPV 16, 18, 31 and 45 are the most popular HPV genotypes in Vietnam. In addition, several molecular modifications—such as PIK3CA, p53 and Kirsten rat sarcoma (KRAS) mutations—have also been observed in small cell neuroendocrine cervical cancer (5). Several studies have demonstrated that the p53 mutation rate in HPV-negative tumours is higher than that found in HPV-positive tumours (6, 7). Furthermore, the presence of p53 mutant forms could increase the risk of malignancy and the metastatic potential of HPV-positive cervical cancers (8). However, the available information about the molecular changes involved in the pathogenesis of neuroendocrine carcinomas of the cervix, especially in association with HPV infection, is limited. It is difficult to propose prognoses, develop targeted therapeutic regimens, and improve outcomes for patients due to the rarity of this disease, as well as the lack of knowledge regarding the distribution of high-risk HPV genotypes and molecular abnormalities.

The aim of this study was to investigate the prevalence and distribution of high-risk HPV genotypes and genetic alterations, as well as the histological and immunohistochemical features of Vietnamese cervical neuroendocrine carcinomas.

## Materials and Methods

### Patients and Tissue Collection

Thirty formalin-fixed paraffin-embedded (FFPE) neuroendocrine cervical tumour tissue blocks were collected from Nov 2016 to May 2018 from the patients who were diagnosed and underwent surgical resection at National Cancer Hospital K in Vietnam. All hematoxylin and eosin (H&E)-stained slides were evaluated by pathologists to confirm the histological diagnosis of neuroendocrine cervical cancer. The clinical characteristics of the patients were obtained from surgical and pathological records. Informed consent was obtained from the patients via a written form and the study was approved by the guidelines of a local ethics committee in Vietnam.

### Immunohistochemistry Analysis

Immunohistochemical staining for p16^INK4a^ was performed using the BenchMark XT system (Ventana Medical System, Tucson, Arizona, USA). The pattern of p16^INK4a^ positivity was scored on a four-tier scale from 0 to 3+ as follows: 0 (no staining in any cell, benign or malignant), 1+ (blush staining in fewer than 10% of tumour cells), 2+ (medium staining in 10%–50% of tumour cells) and 3+ (strong diffuse nuclear and cytoplasmic staining in more than 50% of tumour cells) (9). Tumours scored 2+ and 3+ were considered positive for overexpression.

### DNA Isolation

DNA was isolated using the QIAamp DNA FFPE Tissue Kit (Qiagen, Valencia, CA, USA). The quality of the DNA samples was assessed via polymerase chain reaction (PCR) targeting the *β-globin* gene.

### Assay of HPV Genotyping

The high-risk HPV strains 16, 18, 31 and 45 were detected using PCR with specific primers for each genotype. PCR products were sequenced using the Bigdye Terminator Kit (Applied Biosystems, Foster, CA, USA) to exactly confirm each HPV genotype.

### Analysis of Mutations

Mutations in p53 were examined from exon 5 to exon 8 by directly sequencing PCR products using the Bigdye Terminator Kit (Applied Biosystems, Foster, CA, USA). Epidermal growth factor receptor (EGFR) mutations were detected using the Therascreen EGFR RGQ PCR kit (Qiagen, Valencia, CA, USA). v-Raf murine sarcoma viral oncogene homolog B (BRAF) mutations were detected using the Cobas 4800 BRAF V600 mutation test (Roche, Branchburg, NJ, USA), whereas KRAS mutation were examined using the Cobas KRAS mutation test (Roche, Branchburg, NJ, USA). NRAS mutations were detected using the Therascreen NRAS Pyro Kit 24, V1 (Qiagen, Valencia, CA, USA), based on the pyrosequencing technology of the PyroMark Q24 system.

### Statistical Analysis

The statistical analysis was carried out using SPSS software (IBM Corporation, New York, NY, USA). The potential correlations were analysed using Fisher’s exact test. The statistical significance for all analyses was set at *P* < 0.05.

## Results and Discussion

The HPV DNA of four high-risk genotypes were identified in 26 (86.7%) tumours, of which 17 (56.7%) cases were only infected with one HPV type (single infection), six (20.0%) were co-infected with two HPV types and three (10.0%) were co-infected with three HPV types. HPV 18 was the most common genotype (15 cases, 50.0%), followed by HPV 45 (11 cases, 36.7%), HPV 16 (six cases, 20%) and HPV 31 (six cases, 20%) (Table 1). Accumulated data from previous studies has shown that the rate of HPV in cervical neuroendocrine cancer widely varied from 53% to 100%, depending on the patients’ geographical location and ethnicity (10, 11). HPV16 and HPV18 have been found to be the most predominant genotypes in neuroendocrine carcinomas of the cervix (3, 12–14). Other genotypes—such as HPV31, 45 and 68—are less common (12, 15). However, in the present study, HPV 18 and/or 45 infections were found at a higher frequency (86.7%) in neuroendocrine carcinoma of the cervix, suggesting that HPV 18 and 45 are predominant subtypes in Vietnamese patients with cervical neuroendocrine carcinoma. HPV 31 is a high-risk genotype that is rarely found in cervical cancer in Vietnamese and Asian women (16); however, a relatively high prevalence of HPV 31 (20.0%) in association with neuroendocrine cancer of the cervix was first reported in this study. In agreement with a previous study, there was a high incidence (30.0%) of multiple HPV infections in the present study (11). It has been reported that co-infection with multiple HPV types is likely to increase the risk of high-grade lesions and invasive cancer, implying a synergistic impact on cervical carcinogenesis (17). In addition, the presence of these HPV genotypes was significantly associated with pathological stages (*P* = 0.037), suggesting HPV infection was more commonly found in neuroendocrine cervical cancer with advanced stage disease than in those with early stage disease.

The features of HPV infection in cervical cancer, including cervical neuroendocrine carcinoma, have become increasingly clear, whereas the characteristics of the mutations remain inconclusive. Genetic abnormalities of p53 and NRAS were detected in five (16.7%) and one (3.3%) tumours, respectively, whereas serval mutations—such as EGFR, KRAS, and BRAF—that often occur at a high frequency in many cancers were not found in this study. Out of a total of five p53 mutations identified from exon 5 to exon 8, two of the mutations were found in exon 8 and only one mutation each existed on exons 5, 6 and 7. Five point mutations in the p53 sequence resulted in a single amino acid substitution at codons 140 (Thr-Ile) of exon 5, 205 (Tyr-Cys) of exon 6, 237 (Met-Lys) of exon 7, and 278 (Pro-Ala) and 300 (Pro-Thr) of exon 8. p53 is one of the most frequently mutated tumour suppressor genes observed in many types of human cancer. However, the mutation of the p53 gene is a rare event in carcinomas of the cervix, only accounting for approximately 5% of all cases (18, 19). In this study, p53 mutations were observed to have a relatively high prevalence (16.7%) and were more common in SCNC than in LCNC. A previous study on molecular abnormalities in endocrine tumours of the uterine cervix also indicated a very high frequency of p53 mutations (47.0%) and a higher incidence of p53 gene mutations in SCNC (10). Due to its rarity and small sample size, the reported incidence of p53 mutations in cervical neuroendocrine cancer seems to be highly variable. An NRAS mutation was found in one (3.3%) case, which led to the conversion of Gly into Ser at codon 13 of exon 2. This rate of NRAS mutation was approximately identical with that of a previous report (5). In addition, there was an association between p53 and NRAS mutations (*P* = 0.002), but there was no correlation between HPV infections and p53 or NRAS mutations. In the future, the precise mechanisms of these relationships should be elucidated.

**Figure 1:**
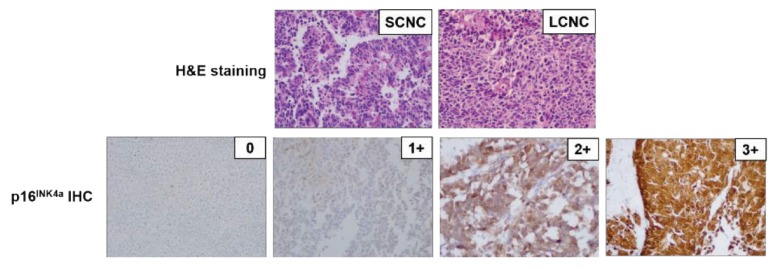
Representative photographs of H&E staining (upper panel) and p16^INK4a^ IHC staining analysis (lower panel). Photographs were taken at 200× magnification SCNC = small cell neuroendocrine carcinoma; LCNC = large cell neuroendocrine carcinoma

Of the 30 cervical neuroendocrine carcinomas, 28 (93.3%) cases were found to be positive for p16^INK4a^ overexpression. Notably, the p16^INK4a^ IHC results showed a significant association with the presence of HPV (*P* = 0.014) (Figure 1), whereas p16^INK4a^ overexpression was not found to be correlated with p53 or NRAS mutation (Table 1). This data suggests that p16^INK4a^ overexpression is significantly correlated with the presence of high-risk HPV strains and might be a biomarker for HPV infection in neuroendocrine carcinoma of the cervix.

## Conclusions

In conclusion, our findings indicate that cervical neuroendocrine carcinomas are universally correlated with HPV infection, especially high-risk types, and a corresponding high frequency of p16^INK4a^ expression. We identified a low prevalence of p53 and NRAS mutations that do not appear to be associated with any specific HPV genotype, whereas the NRAS mutation correlates with the p53 mutation. However, further studies with larger sample sizes should be conducted to confirm the frequency and distribution of genetic alterations, as well as their relationship with HPV infection.

## Figures and Tables

**Table 1:** HPV genotypes, p53 mutation and NRAS mutation, and correlation with clinicopathologic parameters

		HPV		*P*-value	HPV 16		*P*-value	HPV 18		*P*-value	HPV 31		*P*-value	HPV 45		*P*-value	P53		*P*-value	NRAS		*P*-value
		Yes	No		Yes	No		Yes	No		Yes	No		Yes	No		M	W		M	W	
N	30	26	4		6	24		15	15		6	24		11	19		5	25		1	29	
Age				0.602			0.378			0.464			1.000			0.466			0.642			0.467
≤49.3	**16**	13	3		2	14		9	7		3	13		7	9		2	14		0	16	
> 49.3	**14**	13	1		4	10		6	8		3	11		4	10		3	11		1	13	
Subtype				1.000			1.000			0.439			1.000			0.694			0.640			1.000
SCNC	**20**	17	3		4	16		9	11		4	16		9	11		4	16		1	19	
LCNC	**10**	9	1		2	8		6	4		2	8		3	7		1	9		0	10	
Tumors				0.130			0.358			0.060			0.660			0.443			0.622			1.000
Metastasis	**12**	12	0		1	11		9	3		3	9		3	9		1	11		0	12	
Primary	**18**	14	4		5	13		6	12		3	15		8	10		4	14		1	17	
Stage				0.037			0.378			0.060			0.657			1.000			0.157			0.467
I&II	**14**	10	4		4	10		4	10		2	12		5	9		4	10		1	13	
III&IV	**16**	16	0		2	14		11	5		4	12		6	10		1	15		0	16	
P16 IHC				0.014			1.000			0.483			1.000			1.000			0.520			1.000
Positive	**28**	26	2		6	22		15	13		6	22		11	17		5	23		1	27	
Negative	**2**	0	2		0	2		0	2		0	2		0	2		0	2		0	2	

M = mutation; W = wild-type; HPV = human papillomavirus; SCNC = small cell neuroendocrine carcinoma; LCNC = large cell neuroendocrine carcinoma; IHC = immunohistochemistry
